# Anticandidal Activity and Low Cytotoxicity of Modified Analogues of the Tobacco Defensin NaD1

**DOI:** 10.3390/antibiotics14111129

**Published:** 2025-11-07

**Authors:** Olga V. Shevchenko, Ivan V. Bogdanov, Serafima I. Fateeva, Daria N. Melnikova, Anastasia A. Ignatova, Ilia Y. Toropygin, Tatiana V. Ovchinnikova, Ekaterina I. Finkina

**Affiliations:** 1 M.M. Shemyakin and Yu.A. Ovchinnikov Institute of Bioorganic Chemistry, Russian Academy of Sciences, 117997 Moscow, Russiaovch@ibch.ru (T.V.O.); 2 Moscow Center for Advanced Studies, 123592 Moscow, Russia; 3 Institute of Biomedical Chemistry, 119121 Moscow, Russia

**Keywords:** fungal infections, *Candida albicans*, tobacco defensin NaD1, modified analogues of NaD1, antifungal activity, cytotoxicity, zymosan binding, liposome leakage, oligomerization

## Abstract

**Background/Objectives:** The growing resistance development among fungi, including those of *Candida* species, poses significant challenges to public health, emphasizing the need for the implementation of innovative therapeutic approaches. The tobacco defensin NaD1 exhibits a pronounced activity against *C. albicans*, but its relatively high cytotoxicity toward mammalian cells limits its potential application. Here, we investigated anticandidal activity and cytotoxicity of four modified analogues of NaD1 (NaD1-1 T44R/K45R, NaD1-2 L38R, NaD1-3 K36R/L38R, NaD1-4 L38R/T44R/K45R). **Methods:** These peptides contained substitutions with arginine of some amino acid residues in the C-terminal region of NaD1 and in its L5 loop (S_35_KILRR_40_), responsible for the “cationic grip” and binding to phosphatidylinositol 4,5-bisphosphate (PIP4,5), one of the primary targets of tobacco defensin action. **Results:** We showed that the modified NaD1 analogues effectively inhibited the growth of *C. albicans* cells but had a less fungicidal action than NaD1. As compared to NaD1, its modified analogues differed in their sensitivity to the presence of various salts; antifungal activities of NaD1-3 and NaD1-4 were more tolerant to the presence of NaCl and CaCl_2_, respectively. All modified analogues except NaD1-1 did not exhibit hemolytic activity and showed significantly less cytotoxicity towards human immune and epithelial cells compared to NaD1. All modified analogues enhanced the permeability of PIP4,5-containing liposomes, although less effectively than NaD1. Differences in their properties were also demonstrated through experiments on oligomerization and zymosan binding. **Conclusions:** Thus, we proposed that the modified NaD1 analogues NaD1-2, NaD1-3, and NaD1-4 appear to be promising candidate antifungals. However, further in vitro and in vivo studies are required to evaluate their therapeutic potential against critical fungal pathogens.

## 1. Introduction

The global incidence of fungal infections keeps steadily growing, posing a significant risk to immunocompromised populations. Fungal diseases range from common superficial infections to severe invasive fungal affects, which have a lower overall morbidity but are associated with unacceptably high mortality rates despite the application of antifungal therapy [1]. Along with invasive aspergillosis, the *Candida* bloodstream infection and invasive candidiasis pose the greatest threat. According to recent data, approximately 1,565,000 people suffer from *Candida* bloodstream infection or invasive candidiasis each year, with a mortality rate of 63.6% [2]. Clinical treatment of fungal infections is challenging due to the limited number of antifungal drugs available and the increasing prevalence of resistant fungal strains [1]. In view of this, there is an urgent need for the development of innovative antifungal strategies that display principally new molecular mechanisms and provide increased efficacy and selectivity. In this context, plant antimicrobial peptides (AMPs), which act mainly against fungi, can be considered as promising drug candidates. The defensin NaD1, isolated from *Nicotiana alata* flowers, has shown remarkable antifungal properties [3,4].

The tobacco defensin NaD1 has the molecular mass of 5296.32 Da and is characterized by a compact mainly β-sheet structure stabilized by four disulfide bonds that contribute to its high stability and resistance to proteolytic cleavage [5]. This peptide exhibits antibacterial activity only at high concentrations but inhibits the growth of fungi at micromolar concentrations, including clinically important human pathogens such as fungi of the *Candida* genus [6]. Previously, we have shown that NaD1 was fungicidal and equally effective against susceptible and resistant to azoles and echinocandins strains of *C. albicans* [7]. The mechanism of NaD1 action is complex and includes interaction with the components of the fungal cell wall, disruption of membrane integrity, and oxidative damage through the production of ROS and nitric oxide [3,8,9]. It has been demonstrated that phosphatidylinositol 4,5-bisphosphate (PIP4,5) was one of the main targets, binding to which NaD1 formed oligomeric complexes that destabilized the fungal membrane. The peptide dimerization and the formation of so-called “cationic grip” play a key role in this process [10,11]. The multi-target mechanism of action significantly reduces the likelihood of fungal resistance development to this peptide, as it has been demonstrated by resistance induction experiments with *Saccharomyces cerevisiae* and *C. albicans* [12,13]. At the same time, a rather high cytotoxicity of NaD1 toward mammalian cells, whose membranes also contain PIP4,5, and a significant decrease in antifungal activity in the presence of salts at physiological concentrations limit its potential for clinical application [4]. In this regard, the development of NaD1 analogues with improved therapeutic properties is relevant.

Here, we obtained four recombinant modified analogues of NaD1 (NaD1-1 T44R/K45R, NaD1-2 L38R, NaD1-3 K36R/L38R, NaD1-4 L38R/T44R/K45R) containing substitutions with arginine of some amino acid residues in the C-terminal region of the peptide and in the L5 loop responsible for the “cationic grip” and binding to PIP4,5. The previously described chimeric peptide, in which part of the L5 loop was changed (R_35_GFR_38_ instead of S_35_KIL_38_, analogues to the non-binding PIP4,5 tobacco defensin NaD2 [14]), was also synthesized and used in all experiments as a comparison. Antifungal activities of modified analogues were investigated against susceptible and resistant strains of *C. albicans* by microdilution method. The effects of various salts on antifungal activities of these peptides, as compared to NaD1, were examined. Abilities of modified analogues to disrupt the integrity of the fungal cell membrane were evaluated with the use of flow cytometry. Hemolytic activity and cytotoxicity of the peptides were determined using human erythrocytes or human peripheral blood mononuclear cells (PBMCs) and Caco-2 epithelial monolayer, respectively. Abilities of the peptides to cause the permeabilization of PIP4,5 containing liposomes were studied by the calcein release assay. The formation of oligomeric complexes by the peptides in the presence of PIP4,5, as well as their binding to zymosan under saline conditions, were also investigated.

## 2. Results and Discussion

### 2.1. Structural Characterization of Modified Analogues of NaD1

Previously, it has been shown that PIP4,5 binding and the tobacco defensin NaD1 oligomerization play an important role in fungal cell killing and are critical for cytotoxic activity of the peptide [10]. The NaD1 oligomer is formed as a horseshoe-like assembly of seven NaD1 dimers cooperatively binding 14 PIP4,5 molecules. The NaD1 dimer contains a “cationic grip” capable of interacting with anionic headgroups of PIP4,5. The “cationic grip” and interaction with PIP4,5 are carried out by the amino acid residues K4 and H33, as well as by K36, I37, L38, and R40 in the L5 loop (S_35_KILRR_40_) of NaD1 [11]. It has been shown, that NaD1 is also able to interact with phosphatidic acid (PA), forming nearly flat, carpet-like larger oligomeric protein–lipid complexes. These complexes are considered to be weaker compared to the NaD1–PIP4,5 complexes, since PA lacks the inositol head group and S35, K36 I37, L38, R39, and R40 of the peptide take part in PA binding [15].

**Figure 1:**
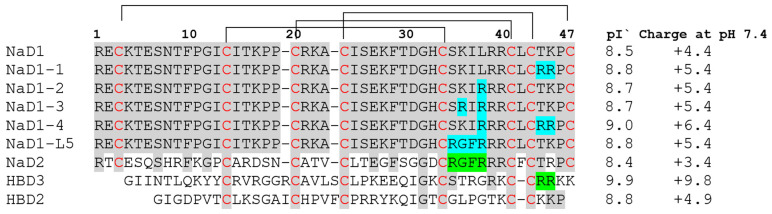
Sequence alignment of the tobacco defensins NaD1 and NaD2, modified analogues of NaD1, human β-defensins, HBD2 and HBD3. Amino acid residues identical to NaD1 are shown in grey. Amino acid substitutions in modified analogues of NaD1 are highlighted in blue. Amino acid residues and sequence fragments borrowed from the structure of other defensins are highlighted in green. The disulfide bonds are marked with square brackets, and cysteine residues are marked in red. The isoelectric points (pI) and charge of the peptides were calculated using the Prot-pi programme tool.

Previously, mutant analogues of NaD1 containing substitutions of the basic amino acid residues from L5 loop, involved in the oligomerization of the peptide and its interaction with target lipids, with alanine or acidic amino acid residues were obtained. Such substitution typically resulted in a decrease in the lipid binding capacity of NaD1 and its cytotoxic properties but also led to a decrease in the antifungal activity of tobacco defensin. For example, the modified analogues NaD1 K36E and NaD1 R39A lost the ability to form oligomers with PA and exhibited reduced antifungal activity against *C. albicans* [15]. The Arg40 substitution with glutamic acid resulted in a decrease, but not a loss, of the peptide ability to bind PIP4,5, but was critical for its oligomerization and the ability to inhibit growth of *F. oxysporum* [11]. A chimeric peptide containing the replacement of the L5 loop part (R_35_GFR_38_ instead of S_35_KIL_38_ by analogy with the tobacco defensin NaD2 (Figure 1) did not bind PIP4,5, and was less active against *Fusarium oxysporum* and tumour cells, as compared to NaD1 [14]. We aimed to develop modified analogues of NaD1 with high antifungal activity and reduced cytotoxicity relative to the tobacco defensin.

Here, we investigated modified analogues of the tobacco defensin NaD1 containing substitutions with arginine of amino acid residues in the L5 loop responsible for the “cationic grip” and binding of PIP4,5 as well as in the nearby C-terminal region of the peptide. It is well known that substitutions with arginine enhance the cationic properties of AMPs that lead to an increased antimicrobial activity by improving their ability to penetrate the cytoplasmic membrane and increasing their tolerance to high salt concentrations [16,17]. We hypothesized that the substitution of various amino acids with arginine may affect the conformation of the “cationic grip” and the affinity of NaD1 for PIP4,5. Nonetheless, these effects might not be crucial for antifungal activity, as they could be offset by additional positive charges and possible changes in the peptide mechanism of action. As is known, some human β-defensins have a spatial structure similar to that of plant defensins and also bind to PIP4,5 [6,18,19]. It has been shown that the C-terminal Arg42 and Arg43, which are absent in the structure of the human β-defensin HBD2, are responsible for a more pronounced antimicrobial activity and a lower sensitivity to the presence of salts of the human β-defensin HBD3 (Figure 1) [20]. Therefore, we obtained the analogue NaD1-1 T44R/K45R, suggesting the possibility that this peptide will be more active than NaD1. The analogues NaD1-2 L38R and NaD1-3 K36R/L38R contained the substitutions in the “cationic grip”. The combined analogue NaD1-4 L38R/T44R/K45R with substitutions both in the C-terminal region of NaD1 and in the L5 loop was also studied (Figure 1). The chimeric peptide R_35_GFR_38_, designated as NaD1-L5, was also obtained and used in all experiments as a comparison (Figure 1).

**Figure 2:**
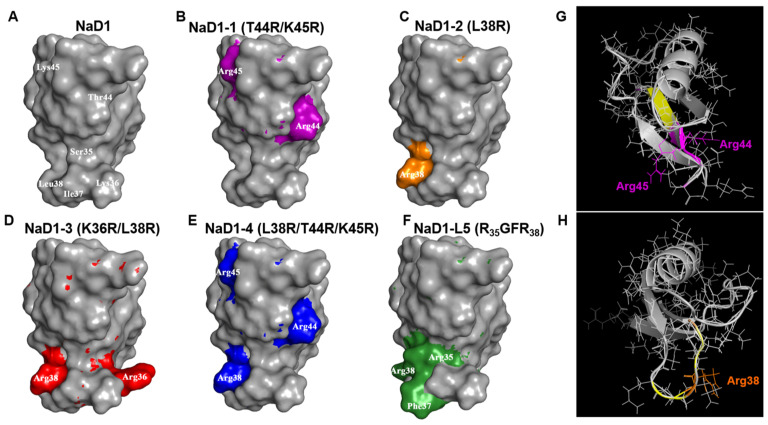
The NMR solution structure of the tobacco defensin NaD1 (PDB 1MR4) (**A**) and structures of its modified analogues built with the bioinformatical tool in PyMOL (**B**–**F**). The replaced amino acid residues in the structures of NaD1-1, NaD1-2, NaD1-3, NaD1-4, and NaD1-L5 are shown in purple, orange, red, blue, and green, respectively. Pymol superposition of NaD1 and NaD1-1 (**G**) or NaD1 and NaD1-2 (**H**) structures; the replaced amino acid residues in NaD1-1 and NaD1-2 are shown in purple (**G**) or orange (**H**), respectively, and other conformational changes are shown in yellow in both cases.

In order to determine the effects of amino acid substitutions on the peptide structure, computer modelling by using the mutagenesis wizard tool in PyMOL was performed. The spatial structure of the tobacco defensin NaD1 (PDB 1MR4), previously solved by NMR in solution, was used as a template in the current study (Figure 2A). Computer visualization of modified analogues of NaD1 demonstrated that all amino acid substitutions had no significant effect on the overall peptide structure (Figure 2A–F). However, the T44R/K45R substitutions in NaD1-1 also caused minor structural changes in the β3-sheet and affected such amino acid residues as Leu42 and Cys43 (Figure 2G). Moreover, even a single L38R substitution in NaD1-2 caused minor conformational changes that also influenced other amino acid residues in the L5 loop including Lys36, Ile37, and Arg39 (Figure 2H). It was also noted that all of the introduced positively charged arginine residues were located on the surface of the peptide structure (Figure 2B–F). Arg44 and Arg45 in the C-terminal regions of NaD1-1 and NaD1-4 were oriented in different directions on the surface of the modified analogues; a similar phenomenon was observed for Arg36 and Arg38 in NaD1-3, which were introduced into the L5 loop (Figure 2B,E). It is worth noting that PyMOL does not modify the underlying scaffold and does not consider steric clashes and therefore may not fully capture all changes in the peptide structure.

**Figure 3:**
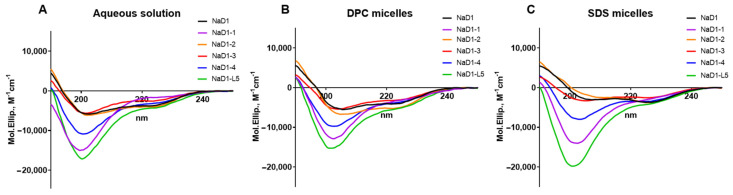
CD-spectra of NaD1 and its modified analogues in aqueous solution (**A**) and in the presence of DPC (**B**) or SDS (**C**) micelles.

The modified analogues NaD1-(1-4) as well as NaD1-L5 were obtained by heterologous expression in *E. coli* cells (Supplementary Materials, Figure S1–S4, Table S1). The secondary structure of NaD1 and its analogues in an aqueous solution and in the presence of DPC or SDS micelles was examined by circular dichroism (CD) spectroscopy (Figure 3, Table 1). The far-UV CD spectra of all peptides in aqueous solution are characterized by a high content of β-sheet structure and a low content of α-helices. No significant differences were observed in the secondary structures of the obtained peptides under these conditions, although NaD1-L5 exhibited a higher percentage of α-helices (Figure 3A, Table 1). However, the secondary structure of NaD1 and its modified analogues changed differently in the presence of detergent micelles. In the presence of zwitterionic DPC micelles, the secondary structures of NaD1 and NaD1-3 remained virtually unchanged, whereas an increase in the proportion of α-helices was found for NaD1-1, NaD1-2, NaD1-4, and NaD1-L5 (Figure 3B). Surprisingly, in the presence of anionic SDS micelles, the proportion of α-helices increased for all peptides, except NaD1-3 for which a small increase in β-sheet content was recorded (Figure 3C). We proposed that the interaction of the NaD1-3 analogue with detergent micelles and cell membranes may occur in a different manner compared to those of the tobacco defensin and other modified analogues possibly due to the presence of Arg36 in its structure (Figure 2D).

It is known that plant defensins, unlike many other AMPs, are characterized by high stability, due to their compact structure stabilized by four disulfide bonds [6]. It was shown that NaD1 retains high antifungal activity after boiling or DTT treatment [13]. In addition, tobacco defensin demonstrates significant resistance to proteolytic enzymes of the human gastrointestinal tract [21]. Here, we investigated the stability of NaD1 analogues by using trypsin and chymotrypsin, digestive enzymes with differing cleavage specificities. For that purpose, peptides were incubated for 24 h at 50 mM ammonium bicarbonate buffer, pH 8.0, with or without protease. All modified analogues showed high stability to trypsin and chymotrypsin digestion for 24 h, similar to that of NaD1 (Supplementary Materials, Figure S5).

### 2.2. Modified Analogues Effectively Inhibit the Growth of C. albicans Cells, but Act Less Fungicidally than NaD1

Using the two-fold serial dilution method, antifungal activities of the obtained analogues were tested against different *C. albicans* strains: susceptible collection strain ATCC 18804; resistant to azoles and anidulafungin collection strain ATCC 10231 and clinical isolates 9.1 and 8.2 [7]. All four analogues NaD1-(1-4) effectively inhibited fungal growth, and minimal inhibitory concentrations (MICs) of 6.25 μM were observed for strains ATCC 18804, ATCC 10231, and 9.1, as in the case of NaD1 (Table 2; Supplementary Materials, Figure S6A–F). The activities of NaD1-3 and NaD1-4 were lower against the resistant strain 8.2, with MIC values twice as high (12.5 μM). At the same time, actions of all analogues except NaD1-1, having no substitutions in the L5 loop, were less fungicidal, and higher minimal fungicidal concentrations (MFCs) were observed. This effect was less pronounced for the peptides NaD1-2 and NaD1-4 for which some MFC values were comparable to those of NaD1 (Table 2). NaD1-L5 was overall the least active of all the peptides tested (Table 2).

Previously, we have shown that NaD1 acted synergistically in combination with the conventional antimycotic caspofungin, which inhibits fungal cell wall biosynthesis, as well as with the membrane-acting human cathelicidin LL-37 [7]. Here we investigated the combined action of NaD1-2, NaD1-3, and NaD1-4 with these antifungals against *C. albicans* ATCC 18804. All three selected analogues acted synergistically with caspofungin, and the FICI value for NaD1-2 was lower than that of the tobacco defensin. At the same time, combinations of all three modified analogues with LL-37 acted less effectively, and only additive effects were observed in the case of NaD1-2 and NaD1-4.

Thus, we demonstrated that four modified analogues NaD1-(1-4), in general, effectively inhibited the growth of both susceptible and resistant strains of *C. albicans*. However, all analogues except NaD1-1, having no substitutions in the L5 loop, involved in the “cationic grip”, are less fungicidal than NaD1. A difference in the effectiveness of some modified analogues against various strains of *C. albicans* unlike NaD1 could be associated with variations in the lipid composition of the cell membranes of these susceptible and resistant fungi [15]. Together with the different potencies of modified analogue combinations with caspofungin and LL-37 compared to NaD1, this fact indicates that the peptides may act differently.

### 2.3. Modified Analogues Differ from NaD1 in Their Sensitivity to Various Salts

The antifungal activity of many AMPs is markedly reduced in the presence of salts at physiological concentrations; however, the underlying mechanisms of this phenomenon remain incompletely understood. Previously, we demonstrated that the anticandidal activity of NaD1 decreased dramatically in the presence of different salts including 150 mM NaCl, 1.25 mM CaCl_2_, and 1.25 mM MgCl_2_ [7]. In this work, we assessed the effects of these salts on the activity of modified analogues of NaD1 against *C. albicans* ATCC 18804 in Sabouraud broth. NaCl was used at concentrations of 50, 100, and 150 mM since its content varies in different biological fluids (for example, the concentration of sodium chloride is much lower in vaginal fluid and saliva than in blood and interstitial fluid [22,23]). We showed that in the presence of 50 mM NaCl the activity of NaD1 and all its modified analogues, except NaD1-3, significantly decreased. The MIC values of NaD1 and NaD1-3 were 50 μM and 12.5 μM, respectively (Table 3; Supplementary Materials, Figure S7). A lower sensitivity of NaD1-3 to the presence of salt was also observed at 100 and 150 mM NaCl (MIC 50 μM), where the activity of other analogues and NaD1 itself was not detected (Table 3). Interestingly, different effects were observed when calcium and magnesium salts were added to the growth medium. All analogues, except for NaD1-1, were less active than NaD1 in the presence of MgCl_2_ (Table 3). At the same time, all analogues in general and especially NaD1-4 (IC_50_ and MIC are 12.5 and 50 μM, respectively) showed greater antifungal activity than NaD1 in a medium containing CaCl_2_ (Table 3). It has been shown that Ca^2+^ and Mg^2+^ ions can have diverse effects on the antimicrobial activity of various AMPs by influencing their interactions with cell membranes, promoting peptide aggregation, or, conversely, stabilizing their conformation [24,25].

Thus, we showed that modified analogues differ from NaD1 in their sensitivity to various salts, suggesting a different mode of action. The antifungal activity of NaD1-3 K36R/L38R decreased significantly less in the presence of sodium chloride at various concentrations than those of all other peptides tested. In our opinion, this correlated with the CD spectroscopy data which showed that NaD1-3 undergoes virtually no structural changes in the presence of zwitterionic and negatively charged micelles unlike all other peptides. We suggested that ionic interactions of NaD1-3 with the fungal cell wall or cell membrane are either stronger or play a less important role in its mechanism of action compared to NaD1 and other modified analogues. To gain insights into the mechanisms of action of the modified analogues of NaD1, we further examined their ability to disrupt the integrity of the fungal cell membrane, induce permeabilization of PIP4,5-containing liposomes, form oligomeric complexes, and bind to zymosan.

### 2.4. All Modified Analogues Are Able to Dimerize and Form Oligomeric Complexes in the Presence of PA and PIP4,5

**Figure 4:**
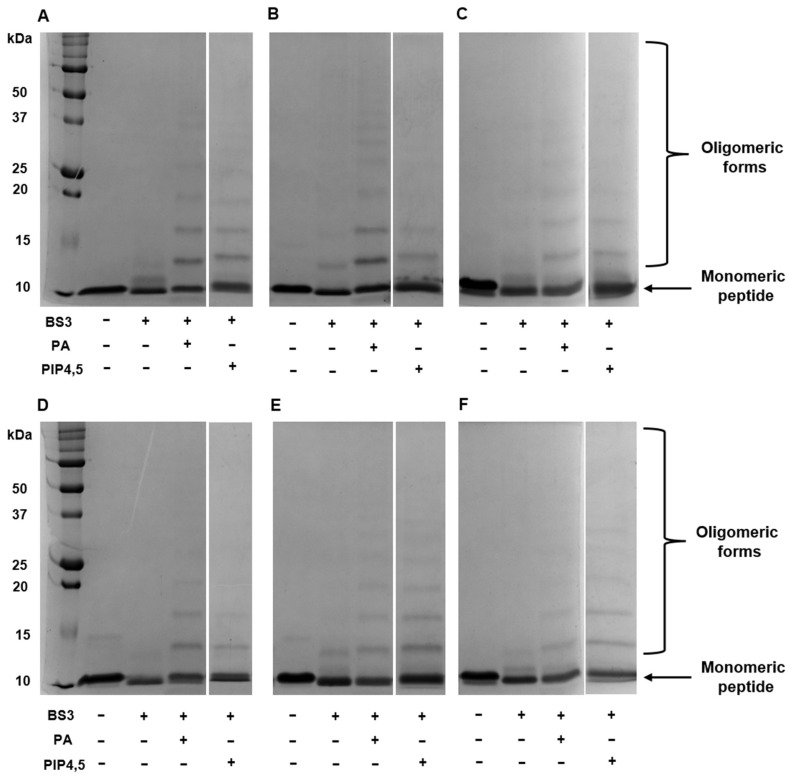
The ability of NaD1 (**A**), NaD1-1 (**B**), NaD1-L5 (**C**), NaD1-2 (**D**), NaD1-3 (**E**), and NaD1-4 (**F**) to form multimeric complexes in the presence of PA or PIP4,5 as determined by protein–protein crosslinking with BS3 followed by SDS-PAGE and Coomassie Brilliant Blue staining. The arrow indicates the monomeric peptide bands. Bands at a higher apparent molecular weight correspond to oligomeric forms of the peptides.

As mentioned above, dimerization and the formation of “cationic grip” play a key role in the binding of PIP4,5 and PA and the subsequent formation of oligomeric protein–lipid complexes. Here, we studied the influence of amino acid substitutions on the ability of modified analogues of NaD1 to form oligomeric complexes with PA and PIP4,5 using the cross-linker bis[sulfosuccinimidyl] suberate (BS3) and reducing SDS-PAGE as described earlier for the tobacco defensin [26]. We showed that in the presence of PA, NaD1 and all its modified analogues, including NaD1-L5, formed large oligomeric assemblies as evidenced by the distinct laddering patterns in the electropherograms (Figure 4A–F). In the presence of PIP4,5, NaD1 and all its modified analogues formed oligomers; however, high-order oligomeric aggregates were less pronounced in some cases, particularly with NaD1-1 and NaD1-2, compared to NaD1 (Figure 4A,B,D). The formation of oligomeric complexes with PIP4,5 also occurred in the case of NaD1-L5, which, as was shown, practically did not bind PIP4,5 in experiments with lipid strips (Figure 4C) [14].

Thus, we concluded that arginine substitutions of amino acids from the C-terminal region of NaD1 and its L5 loop responsible for the “cationic grip” did not result in a loss of the ability of peptides to dimerize and form oligomeric complexes in the presence of lipids. However, in the case of PIP4,5, this effect was less pronounced for some analogues compared to PA.

### 2.5. All Modified Analogues Cause Calcein Leakage from PIP4,5-Containing Liposomes Less Effectively than NaD1

To determine the influence of amino acid substitutions on the ability of modified analogues of NaD1 to cause the permeabilization of artificial membranes, calcein release assays were conducted using liposomes of defined lipid composition. Calcein-loaded PC:PG liposomes in PBS, with or without 5% of PIP4,5 or PA, were used in these experiments. The effectiveness of the peptides was assessed by the release of calcein from liposomes relatively to 0.1% Triton X-100 (Sigma-Aldrich, St. Louis, MO, USA), which was used as positive control. Melittin from bee venom, which exhibits high non-specific membranotropic activity, was used for comparison.

**Figure 5:**
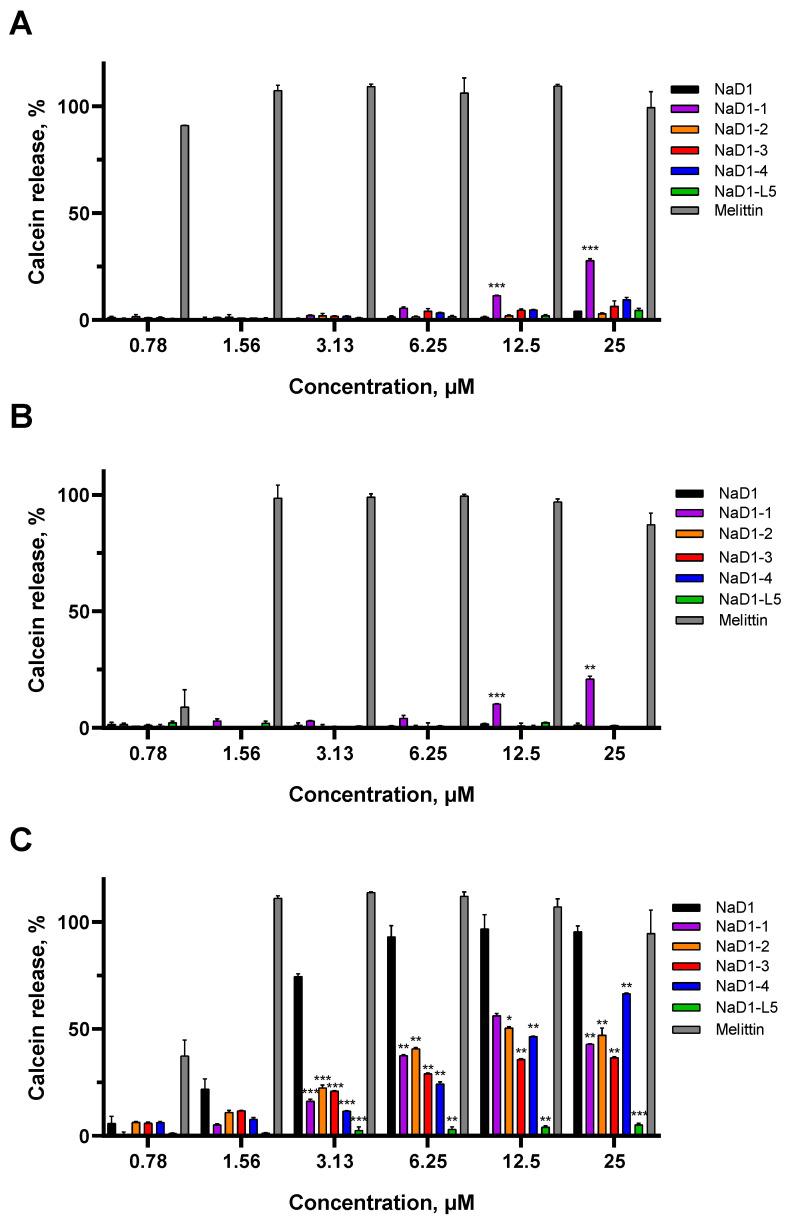
Effects of NaD1 and its modified analogues on the permeability of calcein-loaded liposomes of various compositions: (**A**)—PC:PG liposomes (75:25); (**B**)—PC:PG:PA liposomes (75:20:5); (**C**)—PC:PG:PIP4,5 liposomes (75:20:5). PC—phosphatidylcholine; PG—phosphatidylglycerol; PA—phosphatidic acid; PIP4,5—phosphatidylinositol-4,5-bisphosphate. Error bars represent a standard deviation (±SD) between three technical replications. Significance levels are * *p* ≤ 0.05, ** *p* < 0.01, *** *p* < 0.001. The effects of each analogue on liposomes were compared to those of NaD1 by using an unpaired two-sample *t*-test.

We showed that effects of NaD1 on artificial membranes in PBS which contained NaCl at a concentration of 150 mM were specific, as previously described [10], since the peptide did not affect the PC:PG liposomes without a target lipid and caused almost 100% calcein release at a concentration of 6.25 μM from PIP4,5-containing liposomes (Figure 5A,C). The absence of effects was observed with PA-containing liposomes, although NaD1 forms multimeric complexes with this lipid [15]. Possibly, the lower stability of NaD1–PA complexes compared to those formed with PIP4,5 is the reason for the lack of permeabilizing activity in this case (Figure 5B). In contrast, melittin at a concentration of 1.56 μM induced 100% calcein release from all liposomes tested, regardless of their lipid composition (Figure 5A–C).

All modified analogues except NaD1-1 did not affect PC:PG or PC:PG:PA liposomes like NaD1. NaD1-1 slightly interacted with both PC:PG and PC:PG:PA liposomes and caused the release of approximately 28% and 21% of calcein, respectively, at a concentration of 25 μM (Figure 5A,B). This effect was not observed for NaD1-4 which also contains Arg44 and Arg45 in its C-terminal region. Interestingly, all analogues interacted less efficiently with PIP4,5-containing liposomes than NaD1, with calcein release below 50% at a peptide concentration of 6.25 μM. NaD1-L5, as expected, showed virtually no permeabilizing activity (calcein release below 10% at 25 μM) (Figure 5C) [14]. The reduced ability of NaD1-1, which has no substitutions in the “cationic grip”, to disrupt the permeability of PIP4,5-containing liposomes is consistent with its diminished capacity to form oligomers in the presence of this lipid. Possibly, the T44R/K45R substitutions lead to conformational changes in the NaD1 structure, reducing its ability to oligomerize.

Thus, all modified analogues, except NaD1-1, disrupted the permeability of only PIP4,5-containing liposomes in PBS, similar to NaD1. NaD1-1 also exhibited weak non-specific permeabilizing activity, causing calcein leakage from PC:PG and PC:PG:PA liposomes in the presence of salt. All modified analogues, including NaD1-1, which has no substitutions in the “cationic grip”, caused calcein leakage from PIP4,5-containing liposomes less effectively than NaD1. However, this effect was much less pronounced, as expected, than in the case of NaD1-L5.

### 2.6. All Modified Analogues Disrupt the Integrity of the Fungal Cell Membrane with the Same Efficacy as That of NaD1

Further, we used flow cytometry to compare an ability of modified analogues of NaD1 to affect fungal membrane permeability and kill fungal cells. *C. albicans* ATCC 18804 cells were treated with NaD1 or one of its analogues at MICs (6.25 μM for all peptides) for 4 h, followed by staining with the fluorescent intercalating dye propidium iodide (PI), which penetrates dead or dying cells with damaged membranes. Flow cytometry with PI staining is considered a high-throughput method that allows for the quantification of the effect of antimicrobial peptides (AMPs) on the cell membrane integrity within a large population of cells [27]. The rate of PI uptake during a short incubation time of antimicrobial agent with cells can serve as an indicator to distinguish rapidly membranolytic agents from antibiotics whose lethal effects on membrane integrity are secondary to other targets.

**Figure 6:**
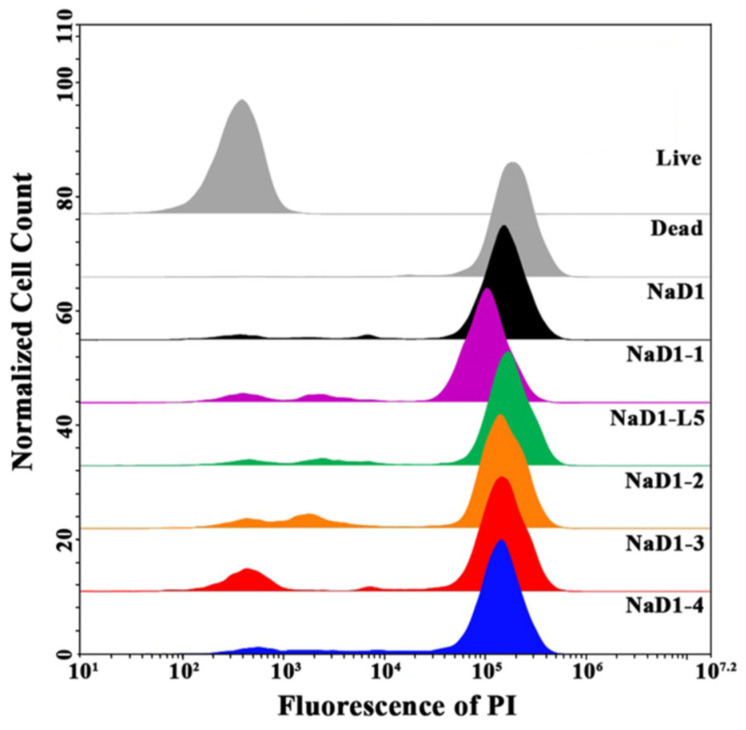
Study of *C. albicans* ATCC 18804 cell viability after incubation for 4 h with NaD1 and its modified analogues, measured by PI uptake using flow cytometry: live and heat-killed cells taken as negative and positive controls, respectively, are shown in grey; cells after incubation with NaD1 and NaD1-1, NaD1-L5, NaD1-2, NaD1-3, and NaD1-4 at MICs are shown in black, purple, green, orange, red, and blue, respectively. Normalized cell count referred to the histogram overlays which were normalized to a 100% scale.

We demonstrated that NaD1 at MIC effectively damaged the fungal cell membrane and killed fungal cells after incubation for 4 h and PI-stained cells accounted for 90.6% (Figure 6; Supplementary Materials, Figure S8). This effect was more pronounced than in the case of incubation for 2 h, when the percentage of PI-stained cells was 52%, as we showed earlier [7]. Surprisingly, the efficiency of all modified analogues of NaD1 was comparable to that of the tobacco defensin, and after 4 h of incubation, the percentages of PI-stained cells were 82.2, 80.1, 80, 85.3, and 87.3% for NaD1-1, NaD1-2, NaD1-3, NaD1-4, and NaD1-L5, respectively (Figure 6; Supplementary Materials, Figure S8).

Our results correlated with previously published data showing that NaD1-L5, with a substitution of the part of the L5 loop, permeabilized *Fusarium oxysporum* hyphae as effectively as NaD1 [14]. Considering that all modified analogues tested here caused calcein leakage from PIP4,5-containing liposome to a lesser extent than NaD1, we suggested that their antifungal activity and the ability to permeabilize *C. albicans* cells may be mediated not only through interaction with PIP4,5.

### 2.7. Modified Analogues of NaD1 Exhibit Varying Affinities for Zymosan Under Saline Conditions

As mentioned above, the mechanism of action of the tobacco defensin NaD1 involves several steps, the first of which is its interaction with the fungal cell wall, followed by the peptide reaching the cell membrane. This peptide binds to fungal cell wall polysaccharides such as β-glucan and, to a lesser extent, chitin. It is proposed that the interaction of NaD1 with negatively charged β-glucan is primarily electrostatic in nature, involving such amino acids as R1, E2, K4, K17, R21, D31, H33, K36, and T44 [8]. It is assumed that salts impair NaD1 interaction with the cell wall, resulting in a significant decrease in its antifungal activity [8].

**Figure 7:**
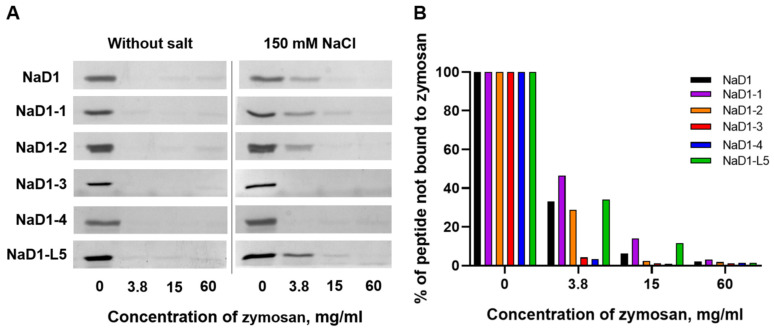
The effect of 150 mM NaCl on zymosan binding to NaD1 and its mutant analogues. (**A**) SDS-PAGE analysis of supernatants from the peptide depletion assays. (**B**) Densitometric analysis of representative gels illustrating the results of incubation of NaD1 and its modified analogues with zymosan in the presence of 150 mM NaCl.

Here we compared the ability of NaD1 and its modified analogues to bind β-glucan in the presence or absence of 150 mM NaCl. Zymosan prepared from yeast cell walls and mainly consisting of β-glucan, and to a lesser extent of mannan, was used in these experiments. The disappearance or weakening of peptide bands on SDS-PAGE suggested efficient peptide binding to insoluble zymosan. We showed that in 10 mM HEPES, pH 7.5, NaD1 and all modified analogues exhibited strong binding capacity to zymosan, as evidenced by the absence of peptide bands after their incubation with insoluble polysaccharides at all concentrations (Figure 7A). In the presence of 150 mM NaCl, the binding efficiency of NaD1, NaD1-1, and NaD1-2, as well as NaD1-L5, was reduced, as indicated by the stronger residual peptide bands at low zymosan concentrations compared to the salt-free condition (Figure 7A,B). At the same time, NaD1-3 and NaD1-4 retained relatively strong binding even under saline conditions, indicating a degree of salt tolerance in their interaction with zymosan (Figure 7A,B). These results for NaD1-3 correlated well with its ability to maintain higher antifungal activity in the presence of NaCl than NaD1 and other modified analogues (Table 3). We proposed that the presence of Arg36 and Arg38, protruding in different directions on the surface of the peptide, determined the salt-tolerant properties of NaD1-3 (Figure 2D). The observed salt tolerance of NaD1-4 upon zymosan binding assay is likely due to its more pronounced cationic properties compared to NaD1 and other modified analogues (pI 9.0 and +6.4 charge at pH 7.4). However, these properties alone probably were not sufficient to confer salt-tolerant antifungal activity.

Thus, we showed that NaD1 and all modified analogues bound to zymosan with high affinity under salt-free conditions. However, in the presence of NaCl, salt-induced inhibition of zymosan binding was observed for NaD1 and several analogues, whereas the affinity of NaD1-3 and NaD1-4 remained comparably high. We hypothesized that these properties may contribute to the relative salt-tolerant antifungal activity of NaD1-3.

### 2.8. All Modified Analogues Except NaD1-1 Are Characterized by the Absence of Hemolytic Activity and Low Cytotoxicity, Unlike NaD1

It is known that tobacco defensin NaD1 exhibits hemolytic and cytotoxic activities [4]. Previously it has been shown that NaD1-L5 exhibits reduced activity against human monocytic lymphoma U937 cells compared to NaD1, suggesting that binding to PIP4,5 is necessary for the potent cytotoxic effects of tobacco defensin [14].

**Figure 8:**
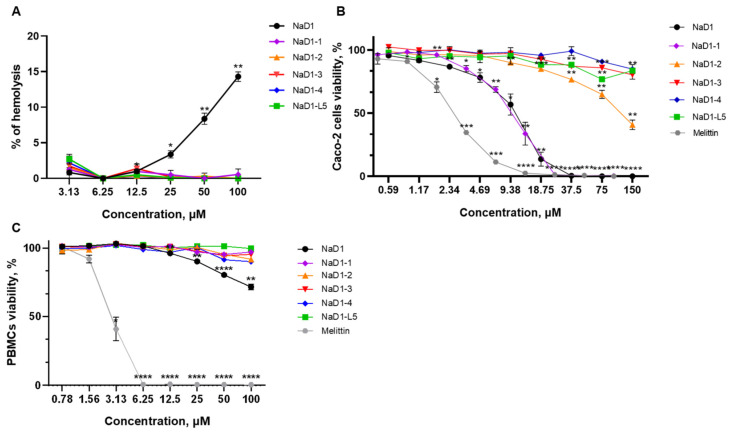
Hemolytic activity (**A**) and cytotoxic effects against a Caco-2 cell monolayer (**B**) or PBMCs (**C**) of NaD1 and its modified analogues. Error bars represent a standard deviation (±SD) between technical replications. The untreated control and samples treated by peptides were compared by an unpaired two-sample *t*-test; significance levels are * *p* < 0.05, ** *p* < 0.01, *** *p* < 0.001, and **** *p* < 0.0001.

Here we examined the ability of modified analogues of NaD1-(1-4) to cause erythrocyte lysis and their cytotoxic effects toward human epithelial and immune cells. Hemolytic activity of NaD1 and its analogues was determined after peptide incubation with freshly isolated human erythrocytes for 2 h. NaD1 at concentrations of 50 and 100 µM caused lysis of about 10 and 15% of erythrocytes, respectively. It is notable that none of the modified analogues caused lysis of erythrocytes, even at a concentration of 100 μM, which is 16 times higher than their average MIC (Figure 8A; Supplementary Materials, Table S2). Under the same conditions, melittin from honeybee venom at 6.25 µM resulted in complete erythrocyte lysis (Supplementary Materials, Table S2).

Cytotoxic activity of the peptides was investigated against human peripheral blood mononuclear cells (PBMCs) and the Caco-2 epithelial monolayer as an in vitro model of the intestinal barrier using a RPMI-1640 medium and resazurin-test. NaD1 demonstrated high toxicity against the Caco-2 monolayer and cell viability was 50 and 0% at its concentrations of 10.5 and 37.5 µM, respectively (Figure 8B; Supplementary Materials, Table S2). These results differed from our previous data where the less pronounced cytotoxic effects of NaD1 against a Caco-2 cell monolayer were demonstrated in DMEM/F12 medium [2]. We showed that NaD1-1 exhibited the same or even slightly more pronounced cytotoxic effect than NaD1 and 50 and 0% cell viabilities were observed at 8 and 25 µM, respectively (Figure 8B; Supplementary Materials, Table S2). It is important to note that three other analogues had much less pronounced cytotoxicity than NaD1. Fifty percent cell viability of Caco-2 cells was observed for NaD1-2 at a high concentration of 120 µM, whereas practically no cytotoxic effects were registered for NaD1-3, NaD1-4, and for NaD-L5 even at a concentration of 150 µM (Figure 8B; Supplementary Materials, Table S2). For comparison, melittin induced approximately 50% and 100% cell death at 2.5 and 12.5 µM, respectively (Figure 8B; Supplementary Materials, Table S2). All modified analogues did not exhibit cytotoxic effects against PBMCs even at a concentration of 100 μM, and only NaD1 inhibited cell viability at high concentrations (cell viability was approximately 72% at a concentration of 100 μM) (Figure 8C; Supplementary Materials, Table S2). The absence of hemolytic activity and cytotoxic effect of NaD1-1 on PBMCs correlated well with its less pronounced ability to disrupt the integrity of PIP4,5-containing liposomes and oligomerize in the presence of this lipid. At the same time, NaD1-1 high toxicity against Caco-2 cells may have been associated with the differences in the lipid composition of human cell membranes and various PIP4,5 content.

Thus, we demonstrated that all modified analogues did not exhibit hemolytic activity unlike NaD1. In addition, all modified analogues except NaD1-1, having no amino acid substitution in the L5 loop, exhibited much less pronounced cytotoxicity than tobacco defensin towards both PBMCs and the Caco-2 epithelial monolayer. It is important to note that an almost-complete absence of cytotoxic effects was observed not only for NaD1-L5, but also for NaD1-3 and NaD1-4, which caused calcein leakage from PIP4,5-containing liposomes.

Based on all the data obtained, we can draw the following conclusions regarding the modified analogues of the tobacco defensin NaD1. The modified analogue NaD1-1, which contains Arg44 and Arg45 in the C-terminal region of the peptide, does not appear promising, as it exhibits anticandidal activity comparable to NaD1, along with low salt tolerance and high cytotoxicity to some human cells. Three other modified analogues of NaD1 contained substitutions with arginine of some amino acid in the L5 loop responsible for the “cationic grip” and binding to PIP4,5 differ from each other, but are all, in our view, promising candidates for further study. NaD1-2, NaD1-3, and NaD1-4 effectively inhibit the growth of susceptible and resistant strains of *C. albicans* and are characterized by the absence of hemolytic activity and much less pronounced cytotoxicity compared to NaD1. In our opinion, an interesting fact is that the antifungal activity of NaD1-3, which contains K36R and L38R substitutions in the L5 loop, is much less affected by the presence of sodium chloride compared to NaD1 and other modified analogues. NaD1-2, NaD1-3, and Nad1-4 bind to PIP4,5 less effectively than NaD1. We suggest that the decreased affinity for the target lipid PIP4,5 leads to a reduction in the fungicidal and cytotoxic activities of these modified analogues of NaD1 but does not critically affect their ability to inhibit fungal growth. The presence of additional arginine residues in their structure may also contribute to this. The observed differences in salt sensitivity, oligomerization ability, zymosan binding, and combined effects with other antifungals among NaD1-2, NaD1-3, and NaD1-4 suggest that their modes of action may involve diverse strategies. Further studies on the effects of these modified analogues on potential intracellular targets and oxidative damage to fungal cells may shed light on this issue.

## 3. Materials and Methods

### 3.1. Materials

The susceptible *C. albicans* collection strain ATCC 18804, resistant to azoles and anidulafungin collection strain ATCC 10231, and resistant to azoles and anidulafungin clinical isolates 9.1 and 8.2 were used in this study [7]. Human colorectal adenocarcinoma Caco-2 cells ATCC HTB-37 were cultured in RPMI-1640 (Corning, Corning, NY, USA) containing 10% fetal bovine serum (FBS; Capricorn Scientific, Ebsdorfergrund, Germany) and a 1× antibiotic–antimycotic (1 × AA) solution (Sigma, St. Louis, MO, USA) at 37 °C in a humidified atmosphere of 5% CO_2_ (CellXpert C170i, Eppendorf, Hamburg, Germany). Peripheral blood mononuclear cells (PBMCs, ATCC PCS-800-011) were also cultured in RPMI-1640 with 10% FBS and 1 × AA at 37 °C in 5% CO_2_.

The tobacco defensin NaD1 (UNIPROT Q8GTM0) was obtained as described previously [28]. Recombinant human cathelicidin LL-37 (UNIPROT P49913) was produced as described in [28] and used together with the conventional antimycotic caspofungin (Sigma, St. Louis, MO, USA) in a checkerboard antifungal assay. Synthetic melittin (purity > 98%) provided by Dr. Sergey V. Sychev from M.M. Shemyakin and Yu.A. Ovchinnikov Institute of Bioorganic Chemistry of the Russian Academy of Sciences were used in cytotoxicity assay.

POPC (1-palmitoyl-2-oleoyl-snglycero-3-phosphocholine), POPG (1-palmitoyl-2-oleoyl-sn-glycero-3-phosphoglycerol), PA (phosphatidic acid), PIP4,5 (phosphatidylinositol (4,5)-bisphosphate) and calcein (Honeywell International Inc., Charlotte, NC, USA) were used in a liposome leakage assay. All lipids were purchased from Avanti Polar Lipids (Alabaster, AL, USA).

### 3.2. Recombinant Production of Modified Analogues of NaD1

The modified analogues of NaD1 with different amino acid substitutions were obtained using a similar approach as previously described for the tobacco defensin [28]. Briefly, the recombinant peptides NaD1-1, NaD1-2, NaD1-3, NaD1-4, and NaD1-L5 were obtained by expression in *Escherichia coli* cells. DNA fragments encoding NaD1 analogues were assembled de novo by PCR with overlapping primers (Supplementary Materials, Figure S1, Table S1) and inserted into the pET-His8-TrxL expression vector. *E. coli* BL-21 (DE3) cells were transformed by plasmid constructs pET-His8-TrxL-NaD1-(1-4,L5) (Supplementary Materials, Figure S2). The recombinant peptides were purified from clarified cell lysate by immobilized metal affinity chromatography (IMAC), followed by dialysis, cleavage of the fusion proteins with cyanogen bromide under acidic conditions, and a second round of IMAC. Final purification of five NaD1 analogues was performed by RP-HPLC. Homogeneity of the obtained peptides were established using SDS-PAGE (Supplementary Materials, Figure S3) and MALDI-TOF mass spectrometry (Supplementary Materials, Figure S4).

### 3.3. Circular Dichroism Spectroscopy

CD spectra of NaD1 and its analogues were recorded using a J-810 spectropolarimeter (Jasco, Hachioji, Tokyo, Japan) at 25 °C in a 0.1 cm path length quartz cell (Hellma GmbH and Co. KG, Mullheim, Germany) in the 190–250 nm range. For that purpose, peptides at a final concentration of 200 μM in aqueous solutions and in detergent micelles of 20 mM dodecylphosphocholine (DPC) (Anatrace, Maumee, OH, USA) or 20 mM sodium dodecyl sulphate (SDS, Sigma-Aldrich, St. Louis, MO, USA) were used. The secondary structure was calculated using the CONTINLL algorithm (CDPro package, Colorado State University, Fort Collins, USA), set of reference spectra: SMP56.

### 3.4. Computational Modelling of the Spatial Structures of NaD1 Analogues

Spatial structures of five modified analogues of NaD1 were built using the mutagenesis wizard tool in PyMOL 1.8.2.0 software (Schrodinger, LLC, New York, NY, USA) based on spatial structure data from the NMR structure of the tobacco defensin NaD1 [PDB ID: 1MR4] [29]. Alterations of the NaD1 surface caused by the introduced amino acid substitutions were coloured and visualized in PyMOL 1.8.2.0.

### 3.5. Antifungal Activity Assay

The antifungal assay was performed by the microdilution method using 96-well microplates and Sabouraud broth as described earlier [7]. Briefly, suspensions of *C. albicans* ATCC 18804, ATCC 10231, 9.1, or 8.2 cells in Sabouraud broth (4 × 10^4^ cells/mL) were mixed with equal volumes of serial two-fold dilutions of NaD1 or its modified analogues in water or caspofungin in 1% DMSO. After that, the plates were incubated at 30 °C for 24 h. A control without antifungals was also tested. Fungal growth was evaluated by measuring absorbance at 630 nm. IC_50_ and MIC were defined as minimal concentrations of antifungal agent, causing 50 and 100% inhibition of fungal growth. The minimum fungicidal concentration (MFC) was defined as the lowest peptide concentration that completely prevented colony formation after plating the contents of the wells with peptide concentrations at MIC and higher onto Sabouraud agar with 2% glucose and 100 μg/mL chloramphenicol (Sigma, St. Louis, MO, USA). To evaluate the impact of salts on the antifungal activity of NaD1 analogues assays in the presence of 50, 100, or 150 mM NaCl, 1.25 mM MgCl_2_ or 1.25 mM CaCl_2_ were carried out. All the experiments were performed twice in triplicate. Microscopic analysis of fungal growth was performed using inverted microscopy (Olympus, Tokyo, Japan). A 0.1% BSA solution was used for the blocking of each microplates well to prevent the adsorption of antifungal agents.

### 3.6. Checkerboard Antifungal Assay

Two-fold serial dilutions of each test agent were prepared in 96-well microplates in water or 1% DMSO in the case of caspofungin and mixed with equal volumes of suspensions of *C. albicans* cells (4 × 10^4^ cells/mL) in Sabouraud broth, followed by 24 h incubation at 30 °C. Controls containing DMSO at a final concentration of 0.5% (cells only and single agent dilutions) were included in all caspofungin combination assays. DMSO at a concentration of 0.5% did not have a noticeable effect on the growth of *C. albicans* cells or on the MIC values of NaD1 and its analogues. To assess the combined effect of antifungal substances, the fractional inhibitory concentration index (FICI) was calculated as FICI = ([A])/([MICA]) + ([B])/([MICB]), where [MICA] and [MICB] are the MICs of the substances A and B, respectively, and [A] and [B] are the concentrations of the substances A and B in iso-effective combinations, respectively.

### 3.7. Zymosan Binding Assay

The polysaccharide-binding activity of NaD1 and its modified analogues was assessed as described previously [30], with minor modifications. The water-insoluble zymosan (Serva, Heidelberg, Germany) prepared from yeast cell walls and mainly consisting of polysaccharide β-1,3-glucan, was washed twice with either 10 mM HEPES, pH 7.5, or 10 mM HEPES, 150 mM NaCl, pH 7.5, and then resuspended in the same buffer at an initial concentration of 60 mg/mL. Then, serial dilutions of zymosan suspension were mixed with NaD1 or its analogues (final concentration of 0.08 mg/mL) and incubated for 1 h with shaking at room temperature. After incubation, the samples were centrifuged at 13,000× *g* for 5 min. The supernatants were mixed with a loading buffer containing 5% β-mercaptoethanol and boiled for 10 min before loading onto SDS-PAGE. Peptide bands were visualized by Coomassie brilliant blue G-250 staining. Gel analysis was performed using Gel Doc XR+ imaging system (Bio-Rad, Hercules, CA, USA) and Image Lab Software v.6.1.0 (Bio-Rad, Hercules, CA, USA).

### 3.8. Flow Cytometry

Membrane permeabilization was quantified by propidium iodide (PI) uptake. *C. albicans* ATCC 18804 cells (2 × 10^4^ cells/mL) in half Sabouraud broth were incubated at 30 °C for 4 h with NaD1 or its modified analogues at MIC. Untreated cells and heat-killed cells (99 °C, 15 min) served as negative and positive controls, respectively. After incubation, the cell suspension was centrifuged at 1600× *g* for 10 min, resuspended in PBS, and incubated with 4 μg/mL PI for 20 min in the dark at room temperature. Samples were analyzed on a NovoCyte 2060R flow cytometer (ACEA Biosciences Inc., San Diego, CA, USA) equipped with blue (488 nm) and red (640 nm) lasers. The obtained data were analyzed using NovoExpress v.1.2.4 software.

### 3.9. Liposome Leakage Assay

The ability of NaD1 and its modified analogues to disrupt artificial liposomes was determined using a calcein release assay mainly as described [10]. Small unilamellar vesicles (SUVs) composed of POPC:POPG (75:25), POPC:POPG:PIP4,5 (75:20:5), or POPC:POPG:PA (75:20:5) were prepared by rehydrating a lipid film with PBS buffer containing 80 mM calcein (pH 7.4). The liposomal suspension was subjected to ten freeze–thaw cycles, followed by extrusion ten times through a 100 nm pore size polycarbonate membrane. Unencapsulated calcein was removed by gel filtration using a Sepharose CL-4B column (GE Healthcare, Chicago, IL, USA). Two-fold serial dilutions of the peptides were added to dye-encapsulated liposomes in PBS in 96-well plates. Calcein release was measured using an Eppendorf Plate Reader AF2200 (Eppendorf, Hamburg, Germany), by recording fluorescence at excitation and emission wavelengths of 485 nm and 535 nm, respectively. Liposome to which PBS (F0) or 0.1% non-ionogenic detergent Triton X-100(Sigma-Aldrich) (Fcontrol) were added were used as negative and positive controls, respectively. Dye leakage (%) was calculated as ((Fsample − F0)/(Fcontrol − F0)) × 100%. Each experiment was independently performed in triplicate.

### 3.10. Dimerization and Oligomerization Assay

NaD1 and its modified analogues (0.5 mg/mL) were incubated with 0.8 mM PA or 0.5 mM PIP4,5 for 30 min at room temperature in 10 mM HEPES, pH 7.5 [15,26]. Lipid-free controls were also used in these experiments. The complexes were then cross-linked by adding 6.25 mM bis[sulfosuccinimidyl]suberate (BS3) (Thermo Scientific, Waltham, MA, USA) for 30 min. After that, the reduced and denatured samples were subjected to SDS-PAGE and stained with Coomassie brilliant blue G-250. Gel analysis was performed using the Gel Doc XR+ imaging system (Bio-Rad, Hercules, CA, USA) and Image Lab Software v.6.1.0 (Bio-Rad, Hercules, CA, USA).

### 3.11. Cytotoxicity Assay

The cytotoxic effects of NaD1 and its modified analogues towards PBMCs (ATCC PCS-800-011) or Caco-2 (ATCC HTB-37) were investigated in 96-well plates using the resazurin method as previously described [28]. Membrane-active peptide melittin from honeybee venom was used for comparison. In brief, Caco-2 cells in a monolayer or 2 × 10^6^ PBMCs per well were incubated with serial dilutions of the peptides for 24 h in RPMI-1640. Resazurin (Sigma, St. Louis, MO, USA) was then added at a final concentration of 70 µM and the plates were incubated for 20 h. Untreated cells (F0) and cells treated by 0.1% Triton X-100 (Sigma-Aldrich) (Fcontrol) were used as negative and positive controls, respectively. Cell viability was assessed by resorufin fluorescence using a 535/595 nm filter and a PlateReader AF2200 as ((Fsample − F0)/(Fcontrol − F0)) × 100%. The CC_50_ was defined as a cytotoxic concentration of the peptide that causes a 50% reduction in cell viability. The experiment was carried out twice in duplicate.

### 3.12. Hemolytic Assay

The hemolytic activity of NaD1 and its modified analogues was assessed in 96-well microplates using fresh human red blood cells (hRBCs), as described [7]. hRBCs were obtained from blood samples collected from healthy donors by certified medical personnel upon informed written consent. All procedures were approved by the Ethics Committee of the Institute of Experimental Medicine (protocol 1/20 of 2/27/2020) and comply with the ethical principles of the Declaration of Helsinki. Melittin was also used in these experiments for comparison. hRBCs (final concentration of 4% *v*/*v*) were mixed with two-fold serial dilutions of the peptides in PBS and incubated for 2 h at 37 °C. Samples were centrifuged to sediment intact red blood cells. Supernatants containing released hemoglobin were transferred to 96-well microplates, and absorbance was measured at 405 nm. Untreated hRBCs in PBS (A0) and hRBC treated with 0.1% Triton X-100 (Sigma-Aldrich) (A_control_) were used as negative and positive controls, respectively. The percentage of hemolysis was calculated as [(A_sample_ − A_0_)/(A_control_ − A_0_)] × 100%. The experiment was carried out twice in duplicate.

### 3.13. Sensitivity to Proteolytic Enzymes

Sensitivity of modified analogues of NaD1 to trypsin or α-chymotrypsin cleavage was investigated comparable to that of NaD1. Enzyme digestion was performed at 50 mM ammonium bicarbonate buffer, pH 8.0, for 24 h at 37 °C using 2.5 ng of trypsin or 10 ng α-chymotrypsin per 1 μg of NaD1 or its modified analogues, as described [31]. The results of proteolysis were monitored by SDS-PAGE (17% T, 3%C). α-Casein, known for its susceptibility to proteolytic digestion, was used in these experiments for comparison.

## 4. Conclusions

In this study, we investigated anticandidal activity and cytotoxicity of four modified analogues of the tobacco defensin NaD1: NaD1-1 T44R/K45R; NaD1-2 L38R; NaD1-3 K36R/L38R; NaD1-4 L38R/T44R/K45R. These peptides contained substitutions with arginine of some amino acid residues in the C-terminal region of NaD1 and in its L5 loop (S_35_KILRR_40_), responsible for the “cationic grip” formation and binding to PIP4,5, one of the primary targets of the tobacco defensin action. We assessed the abilities of the modified analogues to inhibit the growth of both susceptible and resistant strains of *C. albicans*, an influence of various salts on antifungal activity of the peptides, as well as their hemolytic activity and cytotoxic effects on PBMCs and a Caco-2 cell monolayer in comparison with NaD1. We showed that NaD1-1 possessed a pronounced fungicidal action but was also characterized by a high cytotoxicity towards some human cells. Three other analogues, containing substitutions with arginine of some amino acid residues in the L5 loop, effectively inhibited the growth of *C. albicans* cells, but had a lower fungicidal activity than NaD1. The antifungal activity of NaD1-3 and NaD1-4 decreased less in the presence of sodium chloride and calcium chloride, respectively, compared to that of NaD1. NaD1-2, NaD1-3, and NaD1-4 did not exhibit hemolytic activity and showed significantly less cytotoxicity as compared to NaD1. The distinct properties of NaD1-2, NaD1-3, and NaD1-4 were demonstrated through checkerboard assays, experiments on oligomerization in the presence of PIP4,5 and permeabilization of PIP4,5-containing liposomes, as well as on zymosan binding under saline conditions. Taking into account all obtained data, we concluded that three modified analogues NaD1-2, NaD1-3, and NaD1-4 are promising candidates for further study as possible prototypes of new antifungal agents with a high therapeutic index.

## Figures and Tables

**Table 1:** NaD1 and its modified analogues secondary structure estimation (%) predicted from far-UV CD spectra. The main different parameters are highlighted in bold.

Peptide	Condition	α-Helix, %	β-Sheet, %	β-Turn, %	Random, %	NRMSD
NaD1	Aqueous solution	7.5	35.4	21.3	35.8	0.04
	DPC micelles	**9.3**	35.4	21.1	34.2	0.05
	SDS micelles	15.8	31.0	22.2	31.0	0.02
NaD1-1	Aqueous solution	7.6	29.8	23.7	38.9	0.04
	DPC micelles	15.8	30.4	22.6	31.1	0.03
	SDS micelles	16.2	28.5	22.7	32.5	0.02
NaD1-2	Aqueous solution	8.3	34.3	21.2	36.2	0.05
	DPC micelles	15.5	31.1	20.5	32.9	0.03
	SDS micelles	14.2	32.8	21.8	31.2	0.05
NaD1-3	Aqueous solution	5.6	36.8	22.0	35.6	0.05
	DPC micelles	**7.6**	35.8	22.0	34.6	0.04
	SDS micelles	**6.5**	**40.4**	21.3	31.9	0.05
NaD1-4	Aqueous solution	8.9	30.7	22.7	37.7	0.03
	DPC micelles	14.1	31.2	21.7	32.9	0.02
	SDS micelles	16.6	30.8	21.7	31.0	0.03
NaD1-L5	Aqueous solution	**13.5**	22.9	24.3	39.3	0.02
	DPC micelles	18.2	24.1	22.3	35.5	0.02
	SDS micelles	17.8	23.0	23.4	35.9	0.02

**Table 2:** Antifungal activity of NaD1, its modified analogues, and their combinations with other antifungals against different susceptible and resistant *C. albicans* strains in Sabouraud broth. Grey and light green colours indicate antifungal activity that is equal to or slightly higher than that of NaD1, respectively. Yellow, orange, and red colours show activity slightly, strongly, and significantly lower than that of NaD1, respectively.

Peptide	ATCC 18804		ATCC 10231		8.2		9.1		FICI *	
	MIC	MFC	MIC	MFC	MIC	MFC	MIC	MFC	Casp	LL-37
NaD1	6.25	12.5	6.25	12.5	6.25	12.5	6.25	12.5	0.31	0.38
NaD1-1	6.25	12.5	6.25	12.5	nd	nd	6.25	12.5	nd	nd
NaD1-2	6.25	25	6.25	25	6.25	12.5	6.25	12.5	0.26	0.75
NaD1-3	6.25	25	6.25	25	12.5	25	6.25	25	0.38	0.5
NaD1-4	6.25	25	6.25	12.5	12.5	25	6.25	25	0.38	0.63
NaD1-L5	6.25	25	6.25	25	25	>25	6.25	25	nd	nd

nd—not determined; MIC—minimal inhibitory concentration providing 100% inhibition of fungal growth; MFC—minimum fungicidal concentration; FICI—fractional inhibitory concentration index showing the type of action of combined compounds (synergism at FICI ≤ 0.5, additive action at 0.5 < FIC ≤ 1); Casp—caspofungin; *—*C. albicans* ATCC 18804 was used for investigation of combined action of the analogues with other antifungals.

**Table 3:** The influence of different salts on activities of NaD1 and its modified analogues against *C. albicans* ATCC 18804 in Sabouraud broth. Grey colour indicates antifungal activity that is equal to that of NaD1. Light green, deep green, and dark green colours indicate activity that is slightly, strongly, and significantly higher than that of NaD1, respectively. Yellow, orange, and red colours show activity slightly, strongly, and significantly lower than that of NaD1, respectively.

Peptide	No Salts		50 mM NaCl		150 mM NaCl		1.25 mM MgCl_2_		1.25 mM CaCl_2_	
	IC_50_	MIC	IC_50_	MIC	IC_50_	MIC	IC_50_	MIC	IC_50_	MIC
NaD1	1.56–3.13	6.25	12.5–25	50	>50	>50	3.13–6.25	12.5	>50	>50
NaD1-1	1.56–3.13	6.25	12.5–25	50	>50	>50	3.13–6.25	12.5	50	>50
NaD1-2	3.13	6.25	25	>50	>50	>50	6.25–12.5	>50	25	50
NaD1-3	3.13–6.25	6.25	6.25–12.5	12.5	25–50	50	12.5–25	>50	25–50	>50
NaD1-4	3.13–6.25	6.25	25–50	>50	>50	>50	12.5–25	>50	12.5	50
NaD1-L5	3.13–6.25	6.25	25–50	50	>50	>50	12.5–25	50	25–50	50

IC_50_ and MIC—minimal inhibitory concentration providing 50% or 100% inhibition of fungal growth, respectively.

## Data Availability

The original contributions presented in the study are included in the article/Supplementary Materials; further inquiries can be directed to the corresponding author.

## Supplementary Materials

The following supporting information can be downloaded at: https://www.mdpi.com/article/10.3390/antibiotics14111129/s1, Figure S1: Electrophoretic analysis of PCR results for obtained fragments encoding NaD1 analogues; Figure S2: Plasmid vectors pET-His8-TrxL-NaD1-(1-5); Figure S3: Electrophoretic analysis (15% SDS-PAGE) of the purified NaD1 analogues; Figure S4: MALDI mass spectra of the recombinant antimicrobial peptides; Figure S5: SDS-PAGE analysis of stability of NaD1 and its modified analogues; Figure S6: Activity of NaD1 and its modified analogues against *C. albicans* ATCC 18804; Figure S7: The influence of 50 mM NaCl on the activity of NaD1 and NaD1-3 at different concentrations against *C. albicans* ATCC 18804 in Sabouraud broth; Figure S8: Flow cytometry analysis of *C. albicans* ATCC 18804 cell viability after 4 h treatment with tobacco defensin NaD1 and its modified analogues at MIC, assessed by PI uptake; Table S1: List of overlapping primers Table S2: Cytotoxic effects of the tobacco defensin NaD1 and its modified analogues on PBMCs and Caco-2 cells in monolayer.

## References

[1] World Health Organization Antifungal Agents in Clinical and Preclinical Development. Overview and Analysis. https://iris.who.int/bitstream/handle/10665/380498/9789240105140-eng.pdf.

[2] Denning D.W. (2024). Global Incidence and Mortality of Severe Fungal Disease. Lancet Infect. Dis..

[3] Hayes B.M.E., Bleackley M.R., Wiltshire J.L., Anderson M.A., Traven A., van der Weerden N.L. (2013). Identification and Mechanism of Action of the Plant Defensin NaD1 as a New Member of the Antifungal Drug Arsenal against Candida Albicans. Antimicrob. Agents Chemother..

[4] Kerenga B.K., McKenna J.A., Harvey P.J., Quimbar P., Garcia-Ceron D., Lay F.T., Phan T.K., Veneer P.K., Vasa S., Parisi K. (2019). Salt-Tolerant Antifungal and Antibacterial Activities of the Corn Defensin ZmD32. Front. Microbiol..

[5] Lay F.T., Schirra H.J., Scanlon M.J., Anderson M.A., Craik D.J. (2003). The Three-Dimensional Solution Structure of NaD1, a New Floral Defensin from Nicotiana Alata and Its Application to a Homology Model of the Crop Defense Protein AlfAFP. J. Mol. Biol..

[6] Finkina E.I., Shevchenko O.V., Fateeva S.I., Tagaev A.A., Ovchinnikova T.V. (2024). Antifungal Plant Defensins as an Alternative Tool to Combat Candidiasis. Plants.

[7] Shevchenko O.V., Voropaev A.D., Bogdanov I.V., Ovchinnikova T.V., Finkina E.I. (2024). Effects of the Tobacco Defensin NaD1 Against Susceptible and Resistant Strains of Candida Albicans. Pathogens.

[8] Bleackley M.R., Dawson C.S., Payne J.A.E., Harvey P.J., Rosengren K.J., Quimbar P., Garcia-Ceron D., Lowe R., Bulone V., van der Weerden N.L. (2019). The Interaction with Fungal Cell Wall Polysaccharides Determines the Salt Tolerance of Antifungal Plant Defensins. Cell Surf..

[9] Hayes B., Bleackley M., Anderson M., Van der Weerden N. (2018). The Plant Defensin NaD1 Enters the Cytoplasm of Candida Albicans via Endocytosis. J. Fungi.

[10] Payne J.A.E., Bleackley M.R., Lee T.-H., Shafee T.M.A., Poon I.K.H., Hulett M.D., Aguilar M.-I., van der Weerden N.L., Anderson M.A. (2016). The Plant Defensin NaD1 Introduces Membrane Disorder through a Specific Interaction with the Lipid, Phosphatidylinositol 4,5 Bisphosphate. Biochim. Biophys. Acta (BBA) Biomembr..

[11] Poon I.K., Baxter A.A., Lay F.T., Mills G.D., Adda C.G., Payne J.A., Phan T.K., Ryan G.F., White J.A., Veneer P.K. (2014). Phosphoinositide-Mediated Oligomerization of a Defensin Induces Cell Lysis. Elife.

[12] McColl A.I., Bleackley M.R., Anderson M.A., Lowe R.G.T. (2018). Resistance to the Plant Defensin NaD1 Features Modifications to the Cell Wall and Osmo-Regulation Pathways of Yeast. Front. Microbiol..

[13] Finkina E.I., Gerasimova A.A., Shevchenko O.V., Bogdanov I.V., Tagaev A.A., Voropaev A.D., Ovchinnikova T.V. (2025). Modified hevein-like peptide from *Amaranthus caudatus* as a promising agent against pathogenic *Candida* species. Pharmaceutics.

[14] Bleackley M.R., Payne J.A.E., Hayes B.M.E., Durek T., Craik D.J., Shafee T.M.A., Poon I.K.H., Hulett M.D., van der Weerden N.L., Anderson M.A. (2016). Nicotiana Alata Defensin Chimeras Reveal Differences in the Mechanism of Fungal and Tumor Cell Killing and an Enhanced Antifungal Variant. Antimicrob. Agents Chemother..

[15] Järvå M., Lay F.T., Phan T.K., Humble C., Poon I.K.H., Bleackley M.R., Anderson M.A., Hulett M.D., Kvansakul M. (2018). X-Ray Structure of a Carpet-like Antimicrobial Defensin–Phospholipid Membrane Disruption Complex. Nat. Commun..

[16] Cutrona K.J., Kaufman B.A., Figueroa D.M., Elmore D.E. (2015). Role of Arginine and Lysine in the Antimicrobial Mechanism of Histone-derived Antimicrobial Peptides. FEBS Lett..

[17] Olli S., Rangaraj N., Nagaraj R. (2013). Effect of Selectively Introducing Arginine and D-Amino Acids on the Antimicrobial Activity and Salt Sensitivity in Analogs of Human Beta-Defensins. PLoS ONE.

[18] Järvå M., Phan T.K., Lay F.T., Caria S., Kvansakul M., Hulett M.D. (2018). Human β-defensin 2 kills Candida albicans through phosphatidylinositol 4,5-bisphosphate-mediated membrane permeabilization. Sci. Adv..

[19] Phan T.K., Lay F.T., Poon I.K., Hinds M.G., Kvansakul M., Hulett M.D. (2016). Human β-defensin 3 contains an oncolytic motif that binds PI(4,5)P2 to mediate tumour cell permeabilisation. Oncotarget.

[20] Sakagami-Yasui Y., Shirafuji Y., Yamasaki O., Morizane S., Hamada T., Umemura H., Iwatsuki K. (2017). Two arginine residues in the COOH-terminal of human β-defensin-3 constitute an essential motif for antimicrobial activity and IL-6 production. Exp. Dermatol..

[21] Parisi K., Poon S., Renda R.F., Sahota G., English J., Yalpani N., Bleackley M.R., Anderson M.A., van der Weerden N.L. (2020). Improving the Digestibility of Plant Defensins to Meet Regulatory Requirements for Transgene Products in Crop Protection. Front. Plant Sci..

[22] Gonçalves A.C., Marson F.A.D.L., Mendonça R.M.D.H., Ribeiro J.D., Ribeiro A.F., Paschoal I.A., Levy C.E. (2013). Saliva as a Potential Tool for Cystic Fibrosis Diagnosis. Diagn. Pathol..

[23] Tietz K., Klein S. (2018). Simulated Genital Tract Fluids and Their Applicability in Drug Release/Dissolution Testing of Vaginal Dosage Forms. Dissolut. Technol..

[24] Oard S., Karki B. (2006). Mechanism of β-Purothionin Antimicrobial Peptide Inhibition by Metal Ions: Molecular Dynamics Simulation Study. Biophys. Chem..

[25] Jekhmane S., Derks M.G.N., Maity S., Slingerland C.J., Tehrani K.H.M.E., Medeiros-Silva J., Charitou V., Ammerlaan D., Fetz C., Consoli N.A. (2024). Host Defence Peptide Plectasin Targets Bacterial Cell Wall Precursor Lipid II by a Calcium-Sensitive Supramolecular Mechanism. Nat. Microbiol..

[26] Järvå M., Lay F.T., Hulett M.D., Kvansakul M. (2017). Structure of the defensin NsD7 in complex with PIP2 reveals that defensin: Lipid oligomer topologies are dependent on lipid type. FEBS Lett..

[27] O’Brien-Simpson N.M., Pantarat N., Attard T.J., Walsh K.A., Reynolds E.C. (2016). A Rapid and Quantitative Flow Cytometry Method for the Analysis of Membrane Disruptive Antimicrobial Activity. PLoS ONE.

[28] Finkina E.I., Bogdanov I.V., Shevchenko O.V., Fateeva S.I., Ignatova A.A., Balandin S.V., Ovchinnikova T.V. (2024). Immunomodulatory Effects of the Tobacco Defensin NaD1. Antibiotics.

[29] DeLano W.L. (2002). Pymol: An open-source molecular graphics tool. CCP4 Newsl. Protein Crystallogr..

[30] Li H., Kalunke R., Tetorya M., Czymmek K.J., Shah D.M. (2024). Modes of Action and Potential as a Peptide-based Biofungicide of a Plant Defensin MtDef4. Mol. Plant Pathol..

[31] Finkina E.I., Bogdanov I.V., Ignatova A.A., Kanushkina M.D., Egorova E.A., Voropaev A.D., Stukacheva E.A., Ovchinnikova T.V. (2022). Antifungal Activity, Structural Stability, and Immunomodulatory Effects on Human Immune Cells of Defensin from the Lentil *Lens culinaris*. Membranes.

