# Supplementary material for: Anticandidal Activity and Low Cytotoxicity of Modified Analogues of the Tobacco Defensin NaD1
Source: Antibiotics (Basel). 2025 Nov 7;14(11):1129. doi: 10.3390/antibiotics14111129 (PMC12649408; doi:10.3390/antibiotics14111129)
Supplement: Supplementary file 1 [file antibiotics-14-01129-s001.zip › antibiotics-3953266-supplementary.pdf]

Supplementary materials for

Anticandidal activity and Low Cytotoxicity of Modified Analogues of the Tobacco Defensin NaD1

Olga V. Shevchenko[1,2], Ivan V. Bogdanov[1], Serafima I. Fateeva[1], Daria N. Melnikova[1,2], Anastasia A. Ignatova[1], Ilia Y. Toropygin[3], Tatiana V. Ovchinnikova[1,2], Ekaterina I. Finkina[1,2]*

[1] M.M. Shemyakin and Yu.A. Ovchinnikov Institute of Bioorganic Chemistry, Russian Academy of Sciences, 117997 Moscow, Russia; finkina@mail.ru (E.I.F.); contraton@mail.ru (I.V.B.); sh.o.v.2001@gmail.com (O.V.S.); serafima.fateeva@mail.ru (S.I.F.); d_n_m_@mail.ru (D.N.M.); aignatova_83@mail.ru (A.A.I.); ovch@ibch.ru (T.V.O.)

[2] Moscow Center for Advanced Studies, 123592 Moscow, Russia

[3]Institute of Biomedical Chemistry, 119121 Moscow, Russia; toropygin@rambler.ru (I.Y.T.)

* Correspondence: finkina@mail.ru; Tel.: +7-495-335-0900

Materials and Methods

Assembly of genetic construct

DNA fragments encoding five NaD1 analogues were obtained by de novo synthesis using PCR with overlapping primers (Supplementary Materials, Table S1) and Tersus polymerase (Evrogen, Moscow, Russia). The following temperature conditions were selected for PCR: 95°C – 1 min ("hot start"); then 30 amplification cycles (95°C - 30 s, 60°C - 15 s, 72°C - 15 s); further 72°C – 2 min. The quality of the synthesized DNA fragments (197 bp for all peptides) was assessed by electrophoresis in 1.5% agarose gel and ChemiDoc XRS+ gel documentation system (Supplementary Materials, Figure S1).

Each synthesised DNA fragment for each analogue was inserted into the expression plasmid vector pET-His8-TrxL pre-treated with BamHI (Thermo Fisher Scientific, Inc., Waltham, MA, USA). The expression plasmids pET-His8-TrxL-NaD1-(1-4,L5) (Supplementary Materials, Figure S2) were constructed by the ligase-free cloning method using Antarctic Phosphatase (New England Biolabs, Ipswich, MA, USA). Then the reaction mixtures were used to transform competent *E. coli* DH10B cells. Correct plasmid assembly was verified by electrophoresis in 0.8% agarose gel and DNA sequencing performed in two directions.

Heterologous Expression and Purification of Recombinant NaD1 analogues

The *E. coli* BL-21 (DE3) cells were transformed with pET-His8-TrxL-NaD1-(1-4,L5) by heat shock and grown in LB (Luria–Bertani) liquid medium containing 100 μg/ml ampicillin and 20 mM glucose. Cultures were grown to OD_{600} 0.5–0.8 and then induced with 0.2 mM isopropyl-β-D-thiogalactopyranoside (IPTG). Cells grown for 4–6 h at 30 °C were harvested by centrifugation and sonicated in buffer containing 20 mM imidazole. The clarified lysate was applied to a Ni–NTA column, and the bound protein was eluted with 0.5 M imidazole. After dialysis, the fusion protein was dissolved in 80% TFA and cleaved with CNBr. The carrier protein was removed by a second round of Ni–NTA chromatography. Final purification of five NaD1 analogues was performed by RP-HPLC on a semi-preparative Reprosil-Pur C18-AQ column using 0.1% TFA and a 5–80% acetonitrile gradient. Samples homogeneity and molecular identity of recombinant peptides were established using SDS-PAGE (Supplementary Materials, Figures S3), MALDI-TOF mass spectrometry (Supplementary Materials, Figures S4), and circular dichroism (CD) spectroscopy.

Table S1. List of overlapping primers.

NaD1-1:

№	primer sequences (5′-3′)
F1	CCTCGACGCTAACCTGGCCGGATCTATGCGTGAATGCAAAACCGAAAGCAATACAT
F2	GCAAAACCGAAAGCAATACATTCCCGGGCATTTGCATTACCAAACCACCATGCCGTA AAGCTTGTATCAGTGAGAAAT

R1	GCACAGGCAACGACGCAGAATTTTGCTACAATGACCATCGGTAAATTTCTCACTGATA CAAGCTTT
R2	GGTGCTCGAGAGAATTCGCGGATCCTTAGCATGGACGACGGCACAGGCAACGACGCA

NaD1-2:

№	primer sequences (5′-3′)
F1	CCTCGACGCTAACCTGGCCGGATCTATGCGTGAATGCAAAACCGAAAGCAATACAT
F2	GCAAAACCGAAAGCAATACATTCCCGGGCATTTGCATTACCAAACCACCATGCCGTA AAGCTTGTATCAGTGAGAAAT
R4	GCACAGGCAACGACGACGGATTTTGCTACAATGACCATCGGTAAATTTCTCACTGATA CAAGCTTT
R6	GGTGCTCGAGAGAATTCGCGGATCCTTAGCATGGTTTAGTGCACAGGCAACGACGAC

NaD1-3:

№	primer sequences (5′-3′)
F1	CCTCGACGCTAACCTGGCCGGATCTATGCGTGAATGCAAAACCGAAAGCAATACAT
F2	GCAAAACCGAAAGCAATACATTCCCGGGCATTTGCATTACCAAACCACCATGCCGTA AAGCTTGTATCAGTGAGAAAT
R5	GCACAGGCAACGACGACGGATTTGCTACAATGACCATCGGTAAATTTCTCACTGATA CAAGCTTT
R6	GGTGCTCGAGAGAATTCGCGGATCCTTAGCATGGTTTAGTGCACAGGCAACGACGAC

NaD1-4:

№	primer sequences (5′-3′)
F1	CCTCGACGCTAACCTGGCCGGATCTATGCGTGAATGCAAAACCGAAAGCAATACAT
F2	GCAAAACCGAAAGCAATACATTCCCGGGCATTTGCATTACCAAACCACCATGCCGTA AAGCTTGTATCAGTGAGAAAT
R7	GCACAGGCAACGACGGCGAATTTTGCTACAATGACCATCGGTAAATTTCTCACTGATA CAAGCTTT
R8	GGTGCTCGAGAGAATTCGCGGATCCTTAGCATGGACGACGGCACAGGCAACGACGGC

NaD1-L5:

№	primer sequences (5′-3′)
F1	CCTCGACGCTAACCTGGCCGGATCTATGCGTGAATGCAAAACCGAAAGCAATACAT
F2	GCAAAACCGAAAGCAATACATTCCCGGGCATTTGCATTACCAAACCACCATGCCGTA AAGCTTGTATCAGTGAGAAAT
R3	GCACAGGCAACGACGACGAAAACCACGACAATGACCATCGGTAAATTTCTCACTGAT ACAAGCTTT
R6	GGTGCTCGAGAGAATTCGCGGATCCTTAGCATGGTTTAGTGCACAGGCAACGACGAC

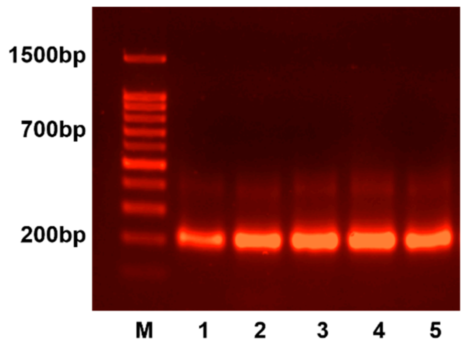

Figure S1. Electrophoretic analysis of PCR results for obtained fragments encoding NaD1 analogues: M – marker of DNA fragment lengths; 1-5 – fragments encoding NaD1-1, NaD1-2, NaD1-3, NaD1-4, and NaD1-L5, respectively.

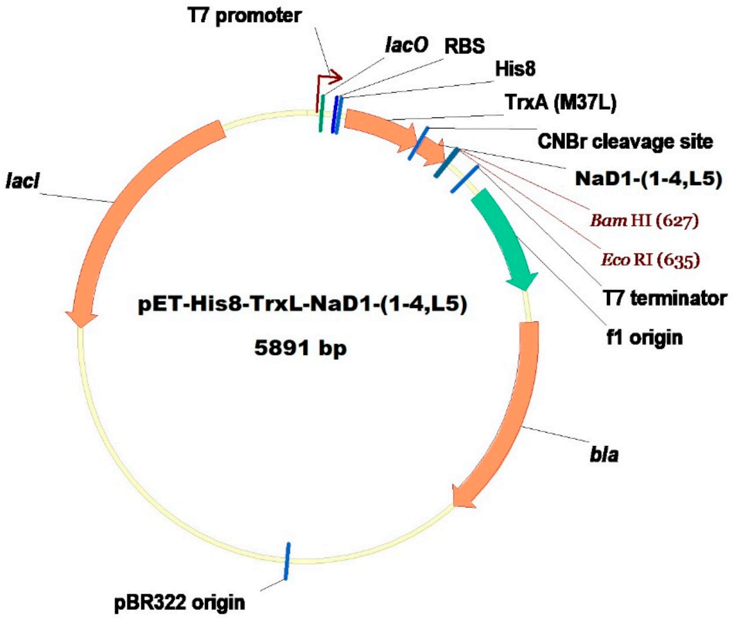

Figure S2. Plasmid vectors pET-His8-TrxL-NaD1-(1-4,L5).

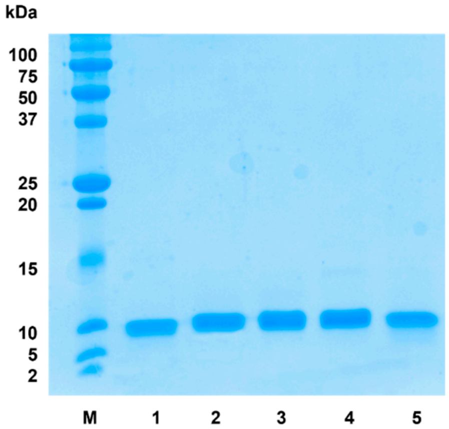

Figure S3. Electrophoretic analysis (15% SDS-PAGE) of the purified NaD1 analogues: M – molecular weight protein standards; 1-5 – purified NaD1-1, NaD1-2, NaD1-3, NaD1-4, and NaD1-L5, respectively.

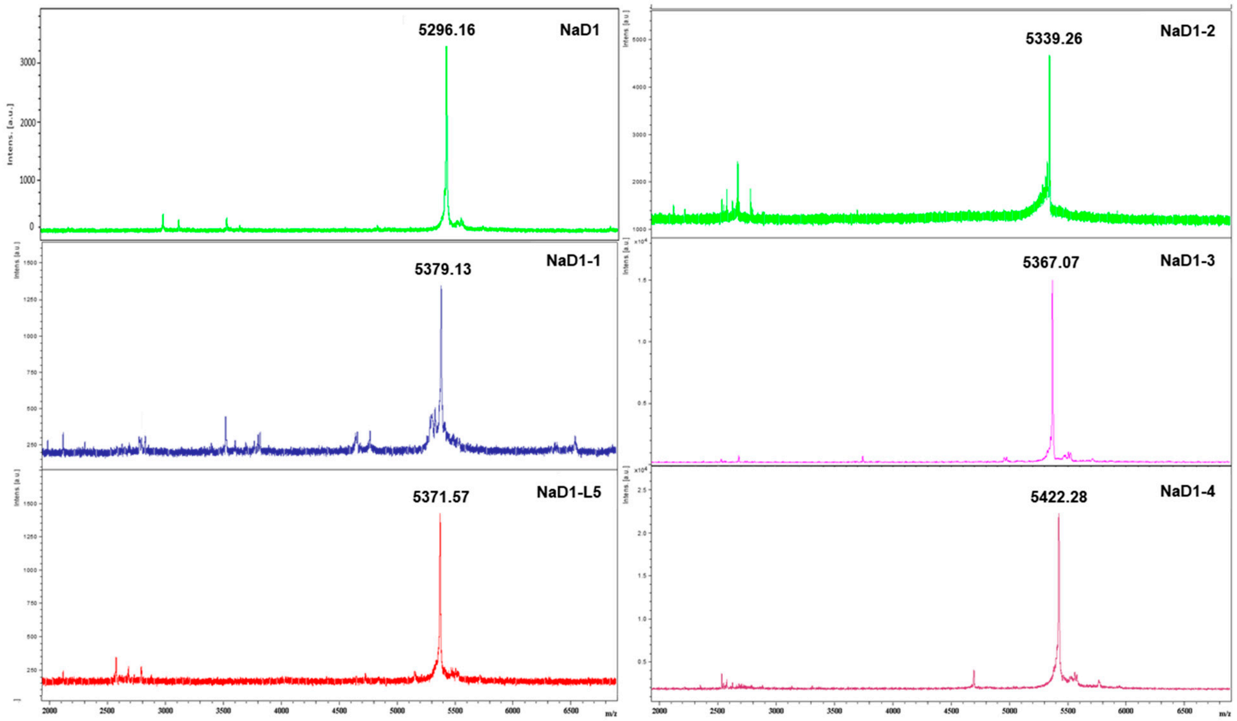

Figure S4. MALDI mass spectra of the recombinant antimicrobial peptides: NaD1 (5296.32 Da); NaD1-1 (5379.42 Da); NaD1-L5 (5371.35 Da); NaD1-2 (5339.35 Da); NaD1-3 (5367.36 Da); NaD1-4 (5422.44 Da). The molecular weights with oxidized S-S bonds were evaluated using the Expasy ProtParam.

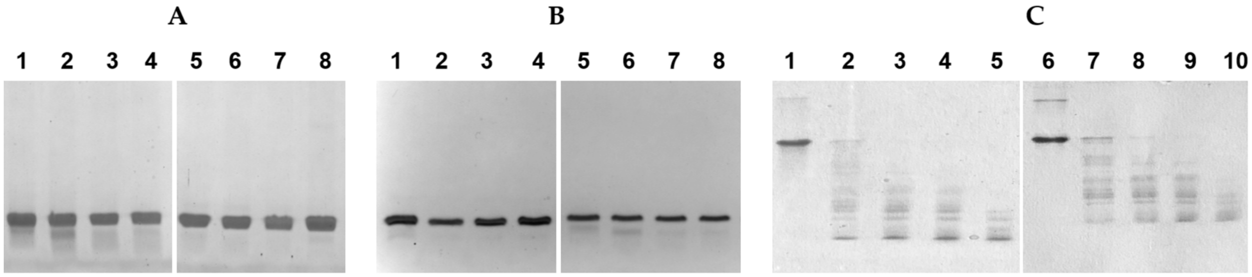

Figure S5. SDS-PAGE analysis of stability of NaD1 and its modified analogues (using analogues NaD1-3 and NaD1-4 as examples). (A) NaD1-3 (1-4) and NaD1-4 (5-8) cleavage by chymotrypsin for 0, 2, 4 and 24 h, respectively. (B) NaD1-3 (1-4) and NaD1-4 (5-8) cleavage by trypsin for 0, 2, 4 and 24 h, respectively. (C) α-Casein cleavage by chymotrypsin (1-5) and trypsin (6-10) for 0, 2, 4, 6 and 24 min.

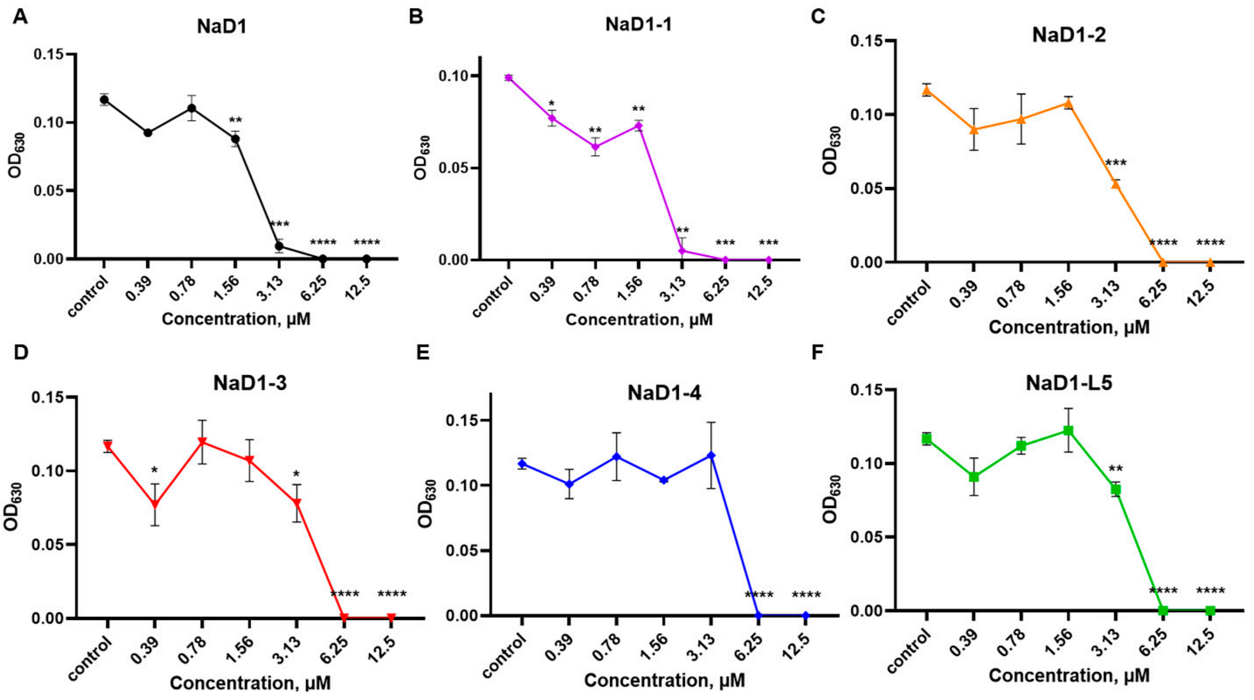

Figure S6. Activity of NaD1 (A) and its modified analogues (B-F) against C. albicans ATCC 18804. Error bars represent a standard deviation (±SD) between technical replications. Significance levels are *p ⩽ 0.05, **p < 0.01, ***p < 0.001, ****p < 0.0001. The untreated controls and samples treated by peptide were compared by unpaired two-sample t-test.

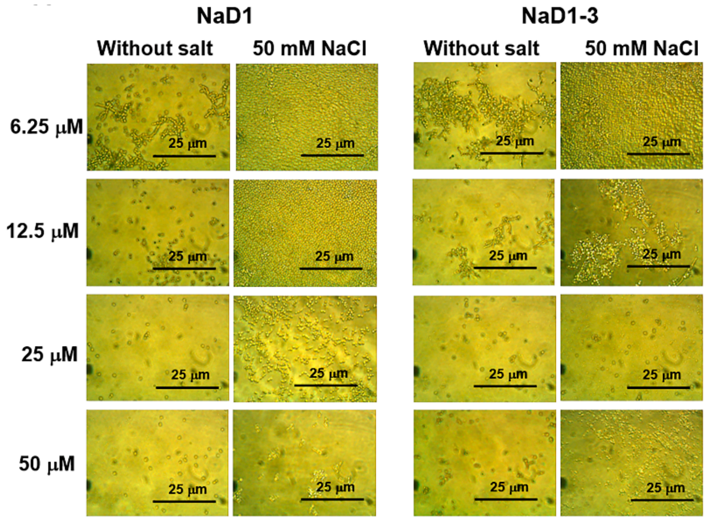

Figure S7. The influence of 50 mM NaCl on the activity of NaD1 and NaD1-3 at different concentrations against C. *albicans* ATCC 18804 in Sabouraud broth (400×magnification).

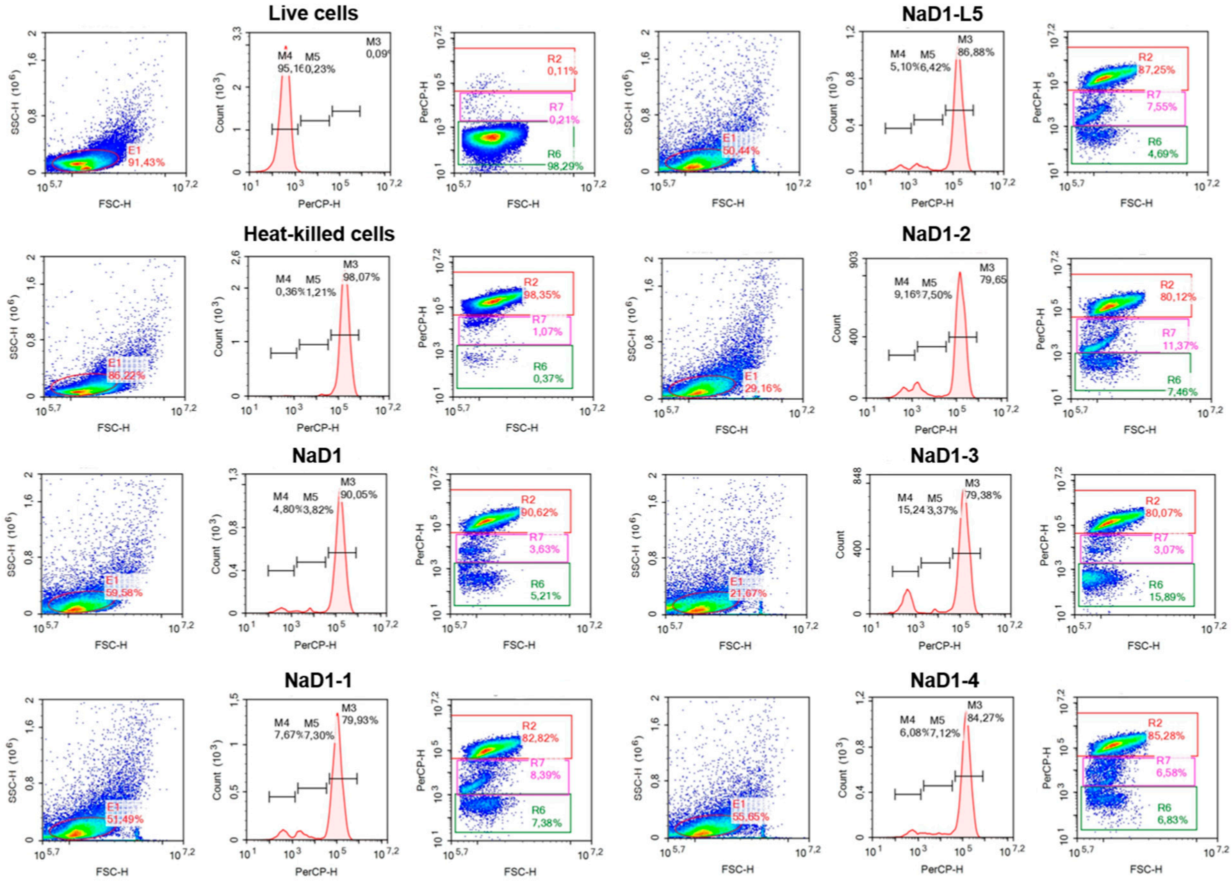

Figure S8. Flow cytometry analysis of *C. albicans* ATCC 18804 cell viability after 4 h treatment with tobacco defensin NaD1 and its modified analogues at MIC, assessed by PI uptake. Live and heat-killed cells were used as negative and positive controls, respectively. Events shown on PI vs. Count and FSC vs. PI plots were gated from the FSC vs. SSC diagram.

Table S2. Cytotoxic effects of the tobacco defensin NaD1 and its analogues on PBMCs and Caco-2 cells in monolayer. Dark green and yellow colors indicate cytotoxic activity that is significantly lower and slightly higher than that of NaD1, respectively.

Peptide	CC_{50}, μM		Hemolysis at 100 μM
	PBMCs	Caco-2	
**NaD1**	>100	10.5	14 %
**NaD1-1**	no effects at concentration of 100 μM	8	no hemolysis
**NaD1-2**		120	
**NaD1-3**		>150	
**NaD1-4**		>150	
**NaD1-L5F**		>150	
**Melittin**	6.10	2.35	100% hemolysis at 6.25 μM
